# Community stigma, victimization, and coping strategies among gay, bisexual, and other cis-gender men who have sex with men in slum communities in Ghana. BSGH-003

**DOI:** 10.1186/s12889-024-18242-1

**Published:** 2024-04-05

**Authors:** Osman Wumpini Shamrock, Gamji Rabiu Abu-Ba’are, Edem Yaw Zigah, Henry Delali Dakpui, Gideon Adjaka, Natalie M. LeBlanc, Amina P. Alio, LaRon Nelson

**Affiliations:** 1 School of Nursing, University of Rochester, Rochester, USA; 2 Behavioral, Sexual and Gender Health Lab, Accra, Ghana; 3 Hope Alliance Foundation, Non-profit Organization, Accra, Ghana; 4 School of Public Health, University of Rochester, Rochester, USA; 5 School of Nursing, Yale University, West Haven, USA; 6 Center for Interdisciplinary Research on AIDS, Yale University School of Public Health, New Haven, Connecticut USA; 7 Behavioral, Sexual and Gender Health Lab, University of Rochester, New Haven, Connecticut USA

**Keywords:** Gay, Bisexual, Cis-gender, Men who have sex with men, Ghanaian slums

## Abstract

**Background:**

Gay, bisexual, and cis-gender men who have sex with men (GBMSM) face severe consequences, especially within stigmatized environments. However, very little is known about the experiences of GBMSM living in slums in SSA and Ghana. This study investigates the experiences of stigma, victimization, and coping strategies and proposes some interventional approaches for combating stigma facing GBMSM in slum communities.

**Methods:**

We engaged GBMSM living in slums in two major Ghanaian cities. We used a time-location sampling and collected data through in-depth individual interviews. Two major themes emerged from the study: (1) insecurities and criminalization of GBMSM activity, and (2) GBMSM coping strategies.

**Results:**

Findings show GBMSM experienced negative attitudes from the community due to their sexual behavior/orientation. GBMSM also developed coping strategies to avert negative experiences, such as hiding their identities/behavior, avoiding gender non-conforming men, and having relationships with persons outside their communities.

**Conclusion:**

We propose interventions such as HIV Education, Empathy, Empowerment, Acceptance, and Commitment Therapy as possible measures to improve the experiences of GBMSM living in Ghanaian slum communities.

## Introduction

In Ghana, research shows that gay, bisexual, and other cis-gender men who have sex with men (GBMSM) experience significant criminalization and stigma from individuals in almost every facet of society [1–3]. Hence, they choose to live a closeted lifestyle by utilizing exceptional discretion and past experiences [4–7]. Currently, Ghanaian law criminalizes same-sex penetrative behavior [8, 9]. The vagueness of the law on sexuality in the country makes its application inconsistent, resulting in exceptional sexual discretion by enforcement agencies [10, 11].

The language used in Ghanaian criminal law contains ambiguity, leading many individuals and law enforcement agents to self-interpret and determine how to apply it to LGBTQI + people [8]. The term “unnatural carnal knowledge” used in the Ghanaian Criminal Code Amendment Act, 2003, does not explicitly mention LGBTQI+; however, it has served to justify the criminal attacks and stigma on members of the groups [8, 12, 13]. According to Atuguba (2019), the law on “unnatural carnal knowledge,” which indicates an unusual form of penetration uncommon in Ghanaian societies, does not extend to sexual desires or expressions. Persons identifying with the LGBTQI + community and not seen engaging in sexual activity involving penetration do not commit offenses connected to the Criminal Code Amendment Act 2003. However, identifying as a member of an LGBTQI + can put an individual at risk of stigma and discrimination [14–17]. A member of parliament in Ghana has proposed an anti-LGBTQI + bill termed “Promotion of Proper Human Sexual Rights and Ghanaian Family Values, 2021,” which proposes to enact legislation to direct the activities of people in the country [18]. The Ghanaian parliament may approve a proposed anti-LGBTQ + bill that criminalizes any persons engaging in LGBTQ+-related activities [19, 20]. At the time of this writing, the Ghanaian parliament is deliberating on passing the Ghanian anti-LGBTQ bill formally named “The Promotion of proper human sexual rights and Ghanaian family values bill,” proposes to among others, criminalize same-sex marriage, same-sex intercourse, intersex, gender-non-conforming behavior, allyship or advocacy for LGBTQ+, with up to five years in prison sentence for offenders [21–23].

While little is known about the experiences of GBMSM in Ghana, the experiences of those living in slum communities remain unaccounted for, yet they face high stigma and negative health outcomes [24]. Slums in Ghana are characterized by unstructured housing settlements, dense population, and poor access to safe drinking water, schools, hospitals, entertainment venues, and transportation [25–27]. Residents within these communities are characterized as violent and having low educational attainment, which is perceived to contribute towards engagement in culturally unacceptable behavior like premarital, extramarital heterosexual sex, and not conforming to heteronormative identities and behavior deemed acceptable by local cultural norms [28]. Coupled with other characteristics, the level of violence in slums can have severe effects and an added safety concern for GBMSM living in these communities.

According to Pebody (2011), GBMSM living in hostile communities such as slums may experience significant violence, harassment, and discrimination from other community members within and external to slum neighborhoods and law enforcement officials [29]. This stigma and discrimination result in adverse health outcomes, including an increased risk of acquiring HIV/STI susceptibility and a lack of engagement in HIV/STI treatment and care [30, 31]. In addition to the legal and social barriers faced by GBMSM, those living in slum communities face multiple economic and social challenges, such as poor living conditions, limited access to healthcare, and limited employment opportunities [30, 32].

In an already stigmatized climate for the LGBTQI + community in Ghana, this study explores how such stigma manifests specifically in the slums for GBMSM [1, 33–38].. The absence of literature on GBMSM living in slums within the Ghanaian context means their experiences related to how others treat them are not fully understood. We address this gap by employing the following methodology and discuss how slum conditions and social behaviors by others may influence GBMSM experiences around crime and stigma.

## Methodology

### Research design

The study employed a qualitative narrative design approach [39–41] to understand the lived experiences of GBMSM in Ghanaian slum communities. The narrative design was a conscious approach to gathering firsthand narratives of how the intersectional stigma of LGBTQ criminalization, poverty and slum community stigma affect GBMSM and the mechanisms men use to cope with these adverse experiences. The qualitative narrative design approach also allowed us to interpret their experiences in the context of the current Ghanaian criminal legal system.

### Study setting

Ghana is located in the Western part of Africa. The dominant religions in the country are Christianity (71%), Islam (18%), and African Traditional Religion/Traditionalist (5%) [42]. The Ghanaian education profile indicates that as of 2020, 71% of children completed primary education, 47% completed lower secondary school, and 35% completed upper secondary school [43]. Studies in Ghana show 52.2% of MSM comprising transgender, gay, bisexual, and straight were educated, with 16.8% with tertiary or higher educational attainment [33]. The same study showed 44.1% of MSM were employed, with 44.1% reporting single or never married [33]. The official language in Ghana is English, however, the indigenous languages are spoken with Twi 16%, Ewe 14%, Fante 11.6%, Boron (Brong) 4.9%, Dagomba 4.4%, Dangme 4.2%, Dagarte (Dagaba) 3.9%, Kokomba 3.5%, Akyem 3.2%, Ga 3.1%, other 31.2% [44]. Slum communities in these two regions were purposefully chosen due to the presence of key population organizations and the ease this provided for the research team when sampling our GBMSM.

### Sampling and recruitment procedure

Recruitment activities were led by our community partner organizations located in Southern Ghana in Accra. To ensure this hard-to-reach population was engaged in data collection, a time-location sampling method was employed. The time-location sampling method has shown to be an effective technique in studying similar population within the LGBTQI + communities [45–47]. The authors purposefully employed this method due to the stigmatizing environment in Ghana for LGBTQI+ [35–38, 48, 49] and enabled researchers to access the sample in safe locations and at optimal times. The method also ensured diversity of participants by providing equal opportunities for inclusion as GBMSM were randomly sampled from multiple visits of the research team to organized monthly meetings GBMSM had with community-based organizations. This sampling method was used to recruit GBMSM in slum communities [50]. Our community-based organizations have been engaged in working with GBMSM in Ghana and have a long history of working with study researchers to recruit and implement studies among GBMSM in the country 1. Community partners screened participants at secured locations to ensure they matched the study criteria for inclusion. Twelve participants were sampled; they reached saturation in responses after the 8th interview. However, community partners continued to interview an additional 4 participants to reach full information saturation.

### Participants

GBMSM participants were at least 18 years and resided in a slum community within Kumasi and Accra, Ghana. Participants age ranged from 18 to 24 years. All participants self-identified as cisgender men having had sexual intercourse with another cis-gender man in the past six months prior to enrollment. For the purpose of this study, we define cis-gender men as individuals whose gender identity corresponds with what was assigned at the time of their birth [51].

### Data collection

#### Procedure

Upon completion of screening, research assistants consented GBMSM to the study, which involved reading the consent form aloud, obtaining signatures as consent to participate in the study, and providing further explanations when needed. Each participant was provided with a copy of the consent form. Research assistants employed in-depth in-person interview to collect data which occurred in anonymous locations to ensure subjects’ identities were always protected. All in-depth interviews were audio were recorded. Four of the 12 interviews were conducted in Twi, a Ghanaian language, as some participants could not effectively express themselves in English; the remainder were in English. Data were collected from participants in January 2022.

#### Nature of questions

The research assistants received training on qualitative interviews using the checklist generated for the study. Consistent with our design, the checklist focused on allowing free and open conversation rather than a typical question-and-respond interview process. Participants were asked to provide personal narratives about their background, experiences of sex, and gender expectations. They also described their experiences of community stigma, openness, and coping strategies about sexuality or sexual behavior and how legislation such as the anti-LGBTQI + bill affected their treatment in their communities.

#### Analytical strategy

Trained research assistants transcribed the audio interview recordings verbatim and deidentified the transcripts by removing specific descriptions that could allow others to identify the participant. We then subjected the transcripts to a multiple-reviewer summative content analysis process [52, 53]. Our team has successfully used this analytical process to understand critical factors in participant accounts [1] We assigned each transcript to at least two reviewers. Each reviewer examined and then reviewed the transcripts to identify the most salient factors raised by the participants, which they independently reported using between 100 and 200 words. The lead author reviewed all summaries and organized the salient points from each write-up into a data spreadsheet, which guided the identification of clusters in the qualitative data and showed the factors that frequently appeared in the transcripts and summaries.

## Results

### Description of participants

Out of 12 GBMSM who participated in the study, two were Muslim, six were Christian, and the remaining four mentioned practicing a mix of Islam and Christianity. Most participants had received formal education. One did not have a Junior High School level education. Six had Senior High School Education, and five had university or tertiary level Education. Six participants indicated they were not employed. The other six were engaged in part-time and full-time jobs.

### Description of themes and categories

Two overarching themes emerged from participant narratives: (1) insecurities and criminalization of GBMSM and (2) GBMSM coping strategies. Within the first theme, two categories emerged: [1] Harm towards GBMSM; and [2] Uncertainty of LGBTQI + bill content and future repercussions for GBMSM (seeking refuge). Categories under the second theme included: [1] Hiding one’s sexuality or conforming to gender norms, and [2] Avoiding gender non-conforming men.

### Insecurities and criminalization of GBMSM

The theme of insecurities and criminalization of GBMSM describes a critical component of GBMSM lives that characterize their interaction with others in their communities and subsequent strategies they employ for anonymity and safety. GBMSM reported that they perceived community members to view them differently as persons who did not conform to the dominant community assigned sex and gender behaviors in the slum, leading to discrimination and marginalization. Within this theme, there were two categories: Harm toward GBMSM and Uncertainty of LGBTQI + bill content and future repercussions for GBMSM (seeking refuge).

### Physical assaults and harm from the community

The hostile attitudes of some slum community members towards GBMSM put them at heightened risk of physical assault. Participants recounted instances where they witnessed other community members physically assaulting their peers because of their sexuality. The fear of bodily harm forced GBMSM to adopt closeted lifestyles, such as associating with GBMSM outside their communities and keeping their sexual behavior/orientation hidden. Speaking on how closeted lifestyles could prevent GBMSM from physical harm, one of the participants mentioned:

I meet my partners secretly, so my sexuality is unknown to my community. Sometimes my friends talk about how they will treat anyone caught in that act. Because I am into guys, I always listen to them closely and ensure they don’t know about my sexuality. (GBMSM Participant)

To avoid physical harm, participants indicated they developed coping strategies to avoid getting caught by others living in the slums. One participant stated:

I always sneak from them (community members), attend to my clients (sex partners), and sneak back to without them knowing them my movement. If they get to know who I am, they can harm me, so I am always careful. (GBMSM Participant)

Another participant mentioned:

I am MSM and prefer that I have my associations outside my community so that when I return home to the community at the end of the day, that would be better. If someone comes into your community to harm you, your community members would disagree because they never see you associating yourself with MSM. It is safer because if you propose to people in your community, and when an issue comes, those people can testify, which would be a problem. So, if I enjoy being outside the community and return when I am okay, it’s better than doing it in my community and facing disgrace later. (GBMSM participant)

Four indicated they had never experienced any form of physical assault or harm from members of their communities. However, past experiences and narrations from others in the LGBTQI + community informed how they navigated the social environment to avoid physical harm. Reported lynching and near-death experiences of other GBMSM manifested mistrust for anyone in the community, including close acquaintances of participants who did not endorse their sexual behavior/orientation. According to one of the participants:

I have never been mistreated or stigmatized, but I have witnessed someone lured to the community, and they almost killed him if not the chief Imam (local religious leader) who came to his rescue. (GBMSM participant)

Another participant mentioned:

I have learned to be extra careful since that incident (violence) because now I know if I should get caught, they will not spare me. So, I am always careful with regard to my sex life. (GBMSM participant)

#### Uncertainty of LGBTQI + bill content and future repercussions for GBMSM

Participants indicated the Ghanaian anti-LGBTQ bill criminalizes their activities, affecting their behaviors and believed it to be contributing to the adverse treatment they receive from others in the slums. Participants also mentioned current and proposed legislation did not support their activities. According to one of the participants:

When I heard about it, I was very scared. I didn’t know what to say because you can’t even tell your friends about it, your straight friends, or those in your community. When you try to defend the community, they will begin to suspect you. So, you have to keep quiet. But within me, I know the bill isn’t going to help us at all. Because there are some people, who are straight men, even though they are girly. Also, some people in the community are transgender or girly. You can’t do anything about their sexuality. If they are walking on the street and you think they are girly, you send them to prison? It will affect our country, productivity, and everything. (GBMSM participant)

Participants stressed the bill violates their fundamental human rights to live freely and would support the legalization of discrimination and marginalization of GBMSM by others whose beliefs depart from those who identify with LGBTQI+. Though the bill is in development, it affects how others treat them in the slums. They also described the Ghanaian government’s role as an institution responsible for preventing the passing of the LGBTQI + bill. According to one of the participants:

It would be nice if the government accepted LGBTQ and gave us our rights. I will be very happy. Because even a man and a woman don’t stand and kiss and have sex; they do it in their rooms. So, you giving us our rights doesn’t mean we are going to do it outside our rooms, so I don’t know why everyone is upset because everyone does their things inside their rooms. The law they want to bring sometimes worries me because I have feminine friends, so sometimes, I ask myself, what if this law is passed? The guys and boys will tag them and set them up (blackmail). Even now that there are no laws, they are setting them up. If it is passed, it will hurt me. (GBMSM participant)

Participants also blamed various media outlets as a medium through which anti- LGBTQI + legislation was propagated. One participant said:

I believe the media feeds society with false information about the community. (GBMSM participant)

### GBMSM coping strategies

The GBMSM in this study reported developing coping strategies to survive the immense marginalization and discrimination they faced in the slum communities. Within this theme were two categories. (1) Hiding sexuality/conforming, and (2) Avoiding gender non-conforming men.

#### Hiding sexuality/conforming

GBMSM physical appearances and behaviors determined how others treated and perceived them within slum communities. Participants reported that people whose behavior and presentations that did not conform to culturally determined gender rules, roles, norms, and expectations of men and women faced greater levels of stigma and resentment from the community.

I have been threatened before. Like when I’m walking, they warn me that if I don’t walk like a man, they will beat me. (GBMSM participant)

It was a girlish guy. If he goes to his partner at night, he will dress like a lady with makeup. They suspected him as MSM in our area because of his behavior. One night, he was seen entering one guy’s place wearing a lady’s nightgown. The guy’s mother saw the girlish guy wearing the nightgown enter her son’s room, so later, she went inside the room to check what they were doing. She saw them in bed having fun and called the area guys. The area guys came and beat both of them. Later, both of them left the community (GBMSM participants).

GBSMSM reported that men who did not exhibit stereotypical feminine behavior did not experience, but rather ascribed to masculine local socially acceptable gender norms adopted certain behaviors to maintain physical safety. Nonetheless, they were extra cautious, not to say they were LGBTQ or MSM, disassociated from non-conforming men, and did not engage in relationships with people who appeared or behaved in a stereotypically feminine manner. According to participants:

They are okay with me because they don’t know anything about my sexuality. So, they treat me normal as any other human being. (GBMSM participant)

I haven’t opened up to anyone in my community about who I am. So, they don’t know. I don’t have feminine features, and I also don’t have feminine friends. And so, they don’t know (GBMSM participant).

#### Avoiding gender non-conforming men

The marginalization and discrimination of GBMSM resulted in a desire to only engage in sexual relationships with persons outside their communities. Participants who wanted to keep appearing as conforming to community-ascribed sex and gender roles and identities chose to associate with sex partners outside the slum community to remain hidden and keep appearing compliant with community norms and expectations. Participants indicated associating with non-gender-conforming men could place one at risk of being suspected of being GBMSM.

I live in Borhu (a slum community), a slum with lots of rough guys. I particularly don’t mingle in my area. I don’t have friends in my community because I don’t like their behavior and lifestyle. (GBMSM participant)

Sometimes, if you work with feminine guys, they think you are also like that. And they will think something bad. I don’t mingle with my community friends. I mingle with outsiders far from my community because it’s my secret (GBMSM participant).

## Discussion

This study’s findings describe the stigma and victimization experiences and coping behaviors of GBMSM who reside in slum communities in Southern Ghana. Prior studies have shown that GBMSM face multiple forms of stigma and marginalization in other countries [54–58]. However, in Ghana, little research has described GBMSM experiences, especially those living in slum communities [1, 59].

The study showed that GBMSM face the threat of physical assault and harm from their communities as some community members physically assaulted them due to their sexual orientation and non-conforming behaviors. Studies have revealed individuals in the LGBTQI + community are four times likely to experience harm from violence [60]. Comparatively, research show the rate of stigma and harm decreases for heterosexual individuals [60, 61]. The findings here align with the global literature that demonstrates, similar to those experiences of GBMSM residing in Ghana’s slums, mistreatment through physical assault and harm to GBMSM are common occurrences [62–64]. Thus, contributing to the evidence that suggests experiences of mistreatment of GBMSM are universal [65].

Within Ghana, research has shown GBMSM face various threats of physical assault and harm from persons with an anti-same-sex intercourse stance [1, 35–38]. Despite interventions proposed by the Ghana Aids Commission to reduce GBMSM stigma through community dialogue, community involvement in stigma and discrimination planning, and awareness raising through traditional leaders [66], the intervention by the commission does not consider the experiences of GBMSM in slums. Also, other interventions that adopt a structural approach to reduce stigma, such as those proposed by (Gyamerah et al., 2020), may present different outcomes when considering the dynamics of living experiences of GBMSM in slum communities to those in urban centers in the country.

We found from this study that the proposed LGBTQI + bill in Ghana facilitated adverse experiences for GBMSM in the slum communities. Though in its development stage, previous research shows that similar bills developed into laws and further criminalized GBMSM. Previous and current laws in other African countries, such as, the Same-Sex Marriage (Prohibition) Act, 2013 of the Republic of Nigeria, the Criminal Code of the Federal Democratic Republic of Ethiopia, Proclamation No. 414/2004 of Ethiopia, the Penal Code of 1998 of Guinea, and the Penal Code Chap. 7:01 Laws of Malawi, and the past Criminal Code of 250 of Mauritius, which was later struck down by the country’s supreme court [67], all detail penalties for persons who do not ascribe to sex-based gender norms and serve as legislative instruments to undermine same-sex activities and relationships [68]. In some African countries such as Uganda and Cameroon these anti-gay legislations direct strict penalties such as life imprisonment, the death penalty for same-sex relations with persons diagnosed with HIV, and the prohibition of LGBTQ in the media spaces [69, 70]. These laws seemingly suggest legislative initiatives in Ghana and in other African countries can be associated with negative consequences for LGBTQ individuals and underscore the challenging and potentially dangerous environments for the community [71].

The study’s findings support existing reports of the role of the media in supporting anti-LGBTQI + and circulating disinformation that contributes to their adverse experiences 10,52. The findings in the study align with the literature that demonstrates media influence on shaping public perception and treatment of LGBTQI + individuals [72–74].

The findings show that socially ascribed gender roles and behaviors were taken seriously among slum community inhabitants. These gender expectations were separated using cues similar to those in Cameroon and South Africa [75], such as behavior and appearance. For locally contextualized non-conforming GBMSM, challenging local (or Westernized) gender/sex binary definitions presented them with potential harm from individuals in the community. As such, some presented themselves as heterosexual men, yet had sex with men anonymously, thus providing further evidence to support the assertions that while one might identify with LGBTQI+, their identity maybe be internalized, and behavior expressed in anonymity [76, 77]. As indicated by participants, the fear of physical assault and harm also manifested in GBMSM living a closeted lifestyle and having sexual relations with others outside their slum communities where they stood the chances of being less recognized, thus reducing the chances of being caught and providing them with the necessary space to operate as GBMSM in communities that were less dense like slums. Whereas we did not find reports from slum communities, previous studies report that generally, GBMSM in Ghana and West Africa who showed mannerisms customarily associated with women were likely to face various forms of discrimination for not conforming to traditional gender-based norms [78–81].

Similarly,Lewis et al. (2023) found that, aside from presenting as conforming to gender and sex-ascribed roles and identities within stigmatized communities, GBMSM sought partners outside their societies to avoid violence and stigma [82]. This study presents a possible third reason: security against threats from people outside their communities who suspect them of engaging in same-sex sexual behaviors. GBMSM used the existing criminal and stigmatized environments as a protective measure. We believe this has not been captured previously in literature among GBMSM living in slum communities in Ghana and is a practice worth noting in literature when understanding coping strategies for these populations.

This study showed GBMSM avoided getting close or forming sexual associations with other GBMSM, particularly gender-nonconforming men in the slums they resided, to avoid being associated with GBMSM and experiencing stigma. While this coping mechanism can protect one from stigma and harassment, it also leads to interpersonal stigma where gender-conforming GBMSM are stigmatized and sometimes assaulted by peers who conform to traditional gender norms [83, 84].To avoid the stressors of being part of a stigmatized group, this finding explicitly shows the extent of personal preservation in the context of internalized homophobia, shame, and guilt toward themselves and other GBMSM [85, 86]. These stressors can lead to other adverse health outcomes for GBMSM such as anxiety, substance abuse, suicide and depression [54, 87]. Highlighting the importance of interventions to address stigma, especially within place and sexuality GBMSM [88, 89].

We identify HIV Education, Empathy, and Empowerment (HIVE^3^), the Dennis Peer Support Model-based approach to guide and develop peer mentoring and support for GBMSM in Ghana [90]. HIVE^3^ and the Dennis Peer Support Model aim to reduce social isolation and stigma and increase peer social support [91, 92]. The intervention, if adequately adopted within the study’s context, will allow members of one group to develop empathy, understanding, and positive attitudes toward another group. We also suggest adopting an Acceptance and Commitment Therapy intervention as adopted by past researchers to understand and create solutions for GBMSM who face significant mental health stress, depression and other forms of social problems due to the stigma and discrimination they face [93–95]. We recommend adopting these interventions to reduce community stigma and victimization for GBMSM in Ghanaian slum communities and promote acceptance, support and empowerment.

### Limitations and future research

The research identified two significant limitations. The first limitation of this study was the absence of comparative literature on GBMSM living in slum communities in Ghana. Given this limitation, it was difficult to determine whether the experiences of GBMSM living in slums have improved or worsened over time. Another associated challenge with the lack of previous literature was whether or not a previous intervention was implemented to curb the adverse experiences of GBMSM living in other Ghanaian slum communities.

The second limitation was the lack of information on GBMSM who moved from urban centers to slums, as well as from rural communities or from other northern parts of Ghana. We propose more studies be conducted on GBMSM living in more Ghanaian slum communities. We also suggest future studies focus on their experiences before they moved to the slums to identify if there are notable differences in experiences for those living in these areas.

In the study, a religious cleric was described to prevent the near-death experience of a GBMSM, which highlights the potential role of religious and traditional leaders in regulating the legal environment within stigmatized communities such as GBMSM in Ghanaian slums. As a result, we recommend future research be conducted to explore this further.

## Conclusion

The study identified two themes that describe the insecurities and criminalization of GBMSM activities and coping strategies. The responses gathered from participant responses show GBMSM in slum communities from within Ghana face significant challenges related to how others treat them and the coping strategies they employ to stay safe. GBMSM in Ghanaian slums showed some innovative techniques in sexual relations by engaging with others outside their communities to reduce the likelihood of stigma or potential harm. GBMSM also revealed they associated with others outside the slums as a way of guaranteeing protection from their slum inhabitants in the event they were accused by outsiders engaged in same-sex sexual behaviors.

The study shows the need to pay attention to the marginalization and discrimination towards GBMSM in Ghanaian slum communities. It is imperative for research moving forward to emphasize the misinformation through media sources as a facilitator of hate sentiments targeted toward GBMSM. Support systems targeted at GBMSM should be appropriately integrated into media discussions when discussing GBMSM topics.

## Data Availability

All data generated or analyzed during this study are included in this published article.

## Abbreviations

GBMSM: Gay, Bisexual, and other Cis-Gender Men Who have Sex with Men
HIVE^3^: HIV Education, Empathy, and Empowerment
